# Fostering idealogical and polical education via knowledge graph and KNN model: an emphasis on positive psychology

**DOI:** 10.1186/s40359-024-01654-4

**Published:** 2024-03-25

**Authors:** Shuangquan Chen, Yu Ma, Wanting Lian

**Affiliations:** 1 School of Marxism, Dalian Maritime University, Liaoning, Dalian, 116000 China; 2 School of Marxism, Dalian University of Technology, Liaoning, Dalian, 116014 China

**Keywords:** Knowledge graph, Educational psychology, Ideological and political education, Relationship prediction, Online education, Mental health

## Abstract

As the primary domain of ideological and political education in higher education institutions, ideological and political courses must align with principles rooted in human psychology and education. Integrating educational psychology into ideological and political teaching in universities enhances the scientific, targeted, and forward-thinking nature of such education. The burgeoning exploration of knowledge graph applications has extended to machine translation, semantic search, and intelligent question answering. Diverging from traditional text matching, the knowledge spectrum graph transforms information acquisition in search engines. This paper pioneers a predictive system for delineating the relationship between educational psychology and ideological and political education in universities. Initially, it extracts diverse psychological mapping relationships of students, constructing a knowledge graph. By employing the KNN algorithm, the system analyzes psychological characteristics to effectively forecast the relationship between educational psychology and ideological and political education in universities. The system's functionality is meticulously detailed in this paper, and its performance is rigorously tested. The results demonstrate high accuracy, recall rates, and F1 values. The F1 score can reach 0.95enabling precise sample classification. The apex of the average curve for system response time peaks at approximately 2.5 s, maintaining an average response time of less than 3 s. This aligns seamlessly with the demands of practical online teaching requirements. The system adeptly forecasts the relationship between educational psychology and ideological and political education in universities, meeting response time requirements and thereby fostering the scientific and predictive nature of ideological and political teaching in higher education institutions.

## Introduction

Nowadays, large-scale online teaching has brought online education to a new level. Facing with the development trend of normalization of online education, how to make use of the intelligent technology and methods to improve the quality of online education to provide guidance for better development of online education is an urgent issue to be solved.

Under the environment of new media communication, various cultural trends of thought have flooded online platforms, which have a serious impact on the psychological and ideological health of young students. However, pure ideological and political education has relatively little impact on students' mental health, and it has become extremely important to integrate psychological education into ideological and political education. The ideological and political course must also follow the laws of human psychology and education [1, 2]. The educational psychology can be applied to the ideological and political teaching, which is helpful to improve the pertinence and predictability of the online education. The integration of these two concepts is conducive to improving the scientific nature of teaching [3, 4]. In the teaching, teachers should adopt different educational methods for different students to improve the pertinence of teaching. In order to better improve the predictability of teaching, we should put ideological and political education in the front to enhance its predictability.

One of the most significant impacts of online education normalization is its potential to democratize access to education. Students from diverse backgrounds and geographical locations can now engage in learning opportunities that were previously limited by physical constraints. This inclusivity fosters a more equitable educational environment, empowering individuals who may have otherwise faced barriers to access. However, this democratization comes with its set of challenges. The lack of face-to-face interaction and the isolation associated with virtual learning can adversely affect student well-being. The absence of social connections and the traditional classroom atmosphere may contribute to feelings of loneliness and disengagement. As such, it is imperative to address these issues to ensure that the benefits of online education are not overshadowed by the potential negative impact on students' mental health. Moreover, the urgency of integrating intelligent technology into virtual learning experiences cannot be overstated. Artificial intelligence (AI) and machine learning algorithms have the potential to personalize education, catering to individual learning styles and pacing. By analyzing students' progress and adapting content accordingly, intelligent technology can create a more tailored and effective learning experience.

The knowledge graph is a concept recently proposed by Google, whose essence is a knowledge base of semantic web [5, 6]. The knowledge graph is a product bred in the semantic web environment, which can be used to describe entity concepts and the logical relationship between them [7, 8]. According to the coverage scope of knowledge, the knowledge graph can be divided into two types, the vertical domain knowledge graph is mainly for a professional field, which mainly pursues the depth and accuracy of knowledge [9]. The open knowledge graph is more like a large encyclopedia database, which contains a variety of knowledge in the real world.

In the context of machine translation, a well-maintained knowledge graph serves as a reservoir of contextual information. The relationships between entities provide crucial context that aids in disambiguating language nuances and improving translation accuracy. For instance, understanding the relationship between a word and its various meanings in different contexts helps refine translations, making them more contextually relevant. Continuous development of the knowledge graph ensures that it stays abreast of evolving language nuances, cultural shifts, and domain-specific terminologies, enhancing the adaptability of machine translation systems over time. Semantic search, on the other hand, relies heavily on the ability to understand the meaning and context behind user queries. A knowledge graph acts as a powerful tool in deciphering these intricacies by establishing connections between entities and capturing the inherent relationships within a vast sea of information. The continuous enrichment of the knowledge graph ensures that semantic search systems can keep pace with evolving user intent, emerging trends, and evolving language usage, thereby delivering more precise and relevant search results.

Now, considering the proposed relationship prediction system, the importance of a robust knowledge graph becomes even more evident. Relationships between entities form the backbone of any predictive model, and a well-maintained knowledge graph provides the necessary foundation. As relationships evolve and new connections emerge, the knowledge graph serves as a living, breathing repository of information. This dynamism enables the relationship prediction system to adapt and improve continually, ensuring its relevance and accuracy over time.

Therefore, the integration of online educational psychology and ideological and political teaching is conducive to improving the scientific nature of online teaching. Before applying educational psychology to the online teaching, it is necessary to understand the relationship between the two concepts. Therefore, it is necessary to design a system that can effectively predict the relationship between the two concepts. The main contributions of this paper are as follows:

- We employed the KNN model to extract the intricate relationship between questionnaires and psychological traits. Leveraging a clustering algorithm, the research delves into a detailed analysis of the psychological characteristics exhibited by students.
- We introduced a systematic approach to predict the relationship between questionnaires and psychological traits and provided a thorough and detailed design of the predictive system's functions, elucidating how it operates in predicting the correlation between questionnaires and psychological characteristics.

## Related works

The knowledge graph assumes a pivotal role in representing the intricate relationships between entities, offering a simulation of the problem-solving approaches employed by human experts [10]. This visual representation, serving as a vital tool, delineates into two distinctive types— the general knowledge graph and the industry knowledge graph. The inception of the knowledge graph concept by Google has catalyzed a surge in scholarly exploration, setting the stage for an intensified research landscape. In recent years, the depth of research on knowledge graphs has notably increased. Scholars have introduced the semantic web in scholarly references, facilitating intelligent communication between humans and machines [11]. An in-depth analysis of students' learning characteristics in online education, grounded in the knowledge graph, is presented, providing a directional compass for the evolution of future educational platforms. Leveraging data mining technology, researchers identify deficiencies within established knowledge graphs, culminating in the formation of cohesive learning clusters [12]. This burgeoning research landscape underscores the evolving significance of knowledge graphs, not merely as visual aids but as dynamic tools shaping the interface between human cognition and computational intelligence, thereby propelling advancements in diverse fields.

In the pivotal stage of preparatory learning, the significance of learning interest and motivation assumes a paramount role in shaping the learning process and outcomes [13, 14]. Contemporary online learning exhibits attributes of heightened psychological immersion, substantial interactivity, and extensive feedback, thereby fostering learner engagement by restructuring the learning trajectory. This approach induces a profound sense of participation and immersion, thereby eliciting learners' interest and galvanizing their motivation to learn. Delving into the psychological facets of students' learning, Frenzel et al. [15] articulate these facets as intrinsic states manifested across various dimensions of students' emotions, perceptions, thoughts, and feelings during mathematical learning. Gojkov et al. [16], in their scrutiny of the learning psychological attributes of exceptional students, conducted an in-depth investigation and interviews, scrutinizing their sentiments, cognitions, perceptions, and emotions throughout the learning process. The analysis encompassed cognitive, non-cognitive, and metacognitive levels, discerning disparities between exceptional students and their counterparts.

In recent years, scholarly attention in the realm of learners' learning psychology has predominantly shifted towards unraveling psychological barriers in the learning process. Investigations have dissected students' learning journeys, accounting for distinct disciplinary characteristics to alleviate or eliminate psychological impediments in learning [17]. The ideological and political course is to make college students form a correct world outlook, and the educational psychology mainly studies the law of psychological activities of teachers and students in school situations. Both these two concepts contain elements such as belief and will [18]. At present, the research on the application of educational psychology has made a lot of research results. The references put forward the theory of constructivism to guide the teaching, which has a profound inspiration for innovative education in ideological and political teaching [19]. By analyzing the characteristics of educational psychology in teaching, [20] proposed that teachers should understand and grasp the psychological characteristics. The effectiveness of ideological and political teaching is studied in the references, the results show that the teaching should integrate personality education theory into the classroom [21]. The teaching strategies of ideological politics are discussed in the references, and the results show that the ideological politics could learn from some theories of educational psychology [22, 23]. Different scholars have analyzed the application of educational psychology, which provides valuable reference for this paper.

## Methodology

### The overall framework of the model

**Fig. 1:**
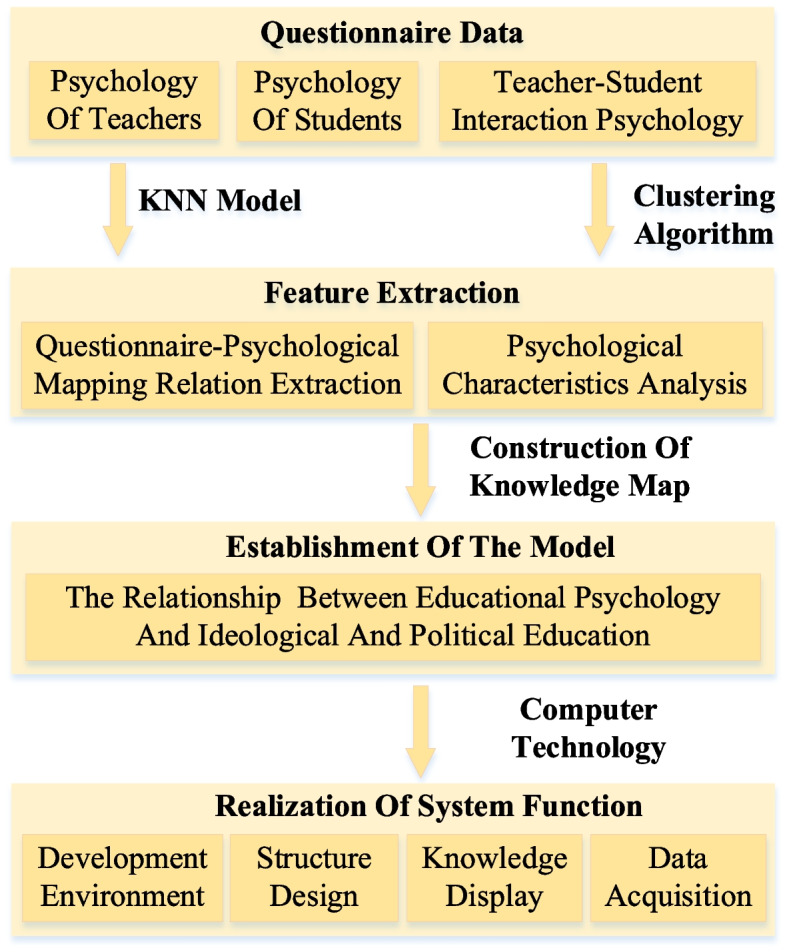
The overall framework of the model

Based on the methods of text classification and cluster analysis, this paper analyzes the mapping relationship between questionnaire and educational psychology. Combined with the questionnaire data, the personal mastery value of students is extracted, which is then transformed into the learning characteristics in different educational psychology. By using the clustering algorithm, students can be divided into several categories according to the differences in learning characteristics. In this paper, the feasibility of clustering algorithm is verified, and the learning characteristics of each class of students are analyzed. The overall framework of the model is shown in Fig. 1.

Prior to integrating educational psychology into teaching, it is imperative to comprehend the nuanced relationship between them. This paper introduces a knowledge graph model grounded in clustering analysis. Within this model, the questionnaire-psychological mapping relationship is derived through a KNN model, and psychological characteristics are scrutinized via a clustering algorithm.

Educational psychology in this model is categorized into three main facets: teacher psychology, student psychology, and the communication psychology between teachers and students. Within the realm of teacher psychology, attributes such as personality charm, teacher prestige, and teaching effectiveness are considered. Student psychology encompasses two key dimensions: self-consciousness and personality tendency. Among these relationships, the teacher-student relationship emerges as the foundational interpersonal dynamic in the teaching context.

### The questionnaire-psychological mapping relation extraction based on KNN

Using the text classification algorithm in text analysis, the k-NN model [24, 25] classifies the questionnaires into different educational psychology firstly. If a questionnaire contains multiple educational psychology, it is impossible to determine the learning characteristics of students in multiple educational psychology, so each questionnaire is considered to be an educational psychology. The data in the questionnaire are analyzed in depth to obtain each the psychological learning characteristics of students. In order to facilitate the analysis, the correlation between the questionnaire and the psychological point is represented by the questionnaire-psychological matrix K, as shown in Formula 1. Formula 1 shows.

1 $$K = \left[ {\begin{array}{*{20}c} {k_{11} } & {k_{21} } & {} & {k_{1n} } \\ {k_{21} } & {k_{22} } & {} & {k_{2n} } \\ {} & {} & {} & {} \\ {k_{m1} } & {k_{m2} } & {} & {k_{mn} } \\ \end{array} } \right]$$

where $k_{mn}$ denotes whether questionnaire m belongs to psychology n, m denotes the number of questionnaires, and n denotes the type of educational psychology. When $k_{mn} = 1$, it means that questionnaire m belongs to psychology n.

The classification model is used to process the extracted text features. Due to the large number of questionnaires and the high dimension of text features, the simplest classification model is selected to deal with the evaluation questions. The extracted text features are obtained by using the k-NN model, and the text features of the test questions are divided into two types. The subordinate relationship between questionnaire data and psychology in training samples is given by authoritative experts in this field. The text features of the training samples have a classification label, each of which represents a psychology.

### The analysis of psychological characteristics based on knowledge graph

The clustering analysis is the process of dividing the set of abstract objects into multiple clusters composed of similar objects. The common clustering analysis algorithms are hierarchical clustering method and DBSCAN density method [26], the DBSCAN density clustering algorithm is selected in this paper, which can divide regions with enough high density into clusters [27].

Combined with text classification, the questionnaire can be classified into educational psychology, and the questionnaire-psychological learning characteristic matrix can be obtained. According to the psychological learning characteristics of each students, the DBSCAN clustering algorithm is used to cluster the students, and the questionnaire-psychological characteristic matrix after clustering can be obtained.

There are three forms of the learning characteristics in educational psychology, which are the high standard, the medium standard and the low standard. $s_{t}$ represents the mastery of educational psychology, as shown in Formula 2.

2 $$s_{t} = (s_{t1} + s_{t2} + \cdot \cdot \cdot + s_{tn} )/h$$

Where $s_{t}$ values in the interval [0, 1]. A higher value indicates a higher degree of mastery in this psychology. If $s_{t} \in [0.7,1]$, it signifies that students have a proficient grasp of this educational psychology, falling into the category of high standards. If $s_{t} \in [0.3,0.7]$, it suggests that students' mastery level in this educational psychology is average, corresponding to the middle standard. If $s_{t} \in [0,0.3]$, it implies that students' mastery level in this educational psychology is not satisfactory, categorizing them under the low standard.

The knowledge graph is composed of entities and the relationships between them, the relationships between entities are the edges in the knowledge graph [28]. Through the clustering analysis of learning characteristics, multiple student classes can be obtained. Then the relationship between students is analyzed, and each student in each category has a learning characteristic value in each psychology. In order to avoid the inaccuracy of the constructed knowledge graph, the authority and rationality of the model are verified according to the difference of learning stability [29].

In order to verify the effect of knowledge graph, the stability of educational psychology is introduced, and its calculation is shown in Formula 3.

3 $$Mu_{tx} = (sk_{tx} - ck_{zx} )^{2}$$

Where $Mu_{tx}$ represents the stability value of educational psychology, which indicates the learning characteristics of students in educational psychology.

The educational psychological balance is introduced to evaluate the model, and its calculation is shown in Formula 4.

4 $$Mu_{t} = \frac{{\sum\limits_{1}^{n} {Mu_{tx} } }}{n}$$

Where $$Mu_{t}$$$$M{u}_{t}$$ represents the variance between all learning characteristics and the learning characteristics of the student.

### Prediction system design

The knowledge graph model based on cluster analysis is introduced above, which can effectively predict the relationship between these two concepts. Then, the system module will be designed in detail from four aspects, which are the software development environment and experimental data, the structure design, the data acquisition module and the knowledge display and storage.

The background language of the system is Python that has low learning cost and many mature frameworks, and the Django framework of Python language is used in this paper. According to the size and functional requirements of system, the Django framework is selected in this paper. The database selected in this paper is ProQuest Psychology Database, which is the most comprehensive full-text database of full-text journals of pedagogy and psychology in the world. The data used in this paper includes two parts, one is the basic information data of students, the other is the answer record data of students.

**Fig. 2:**
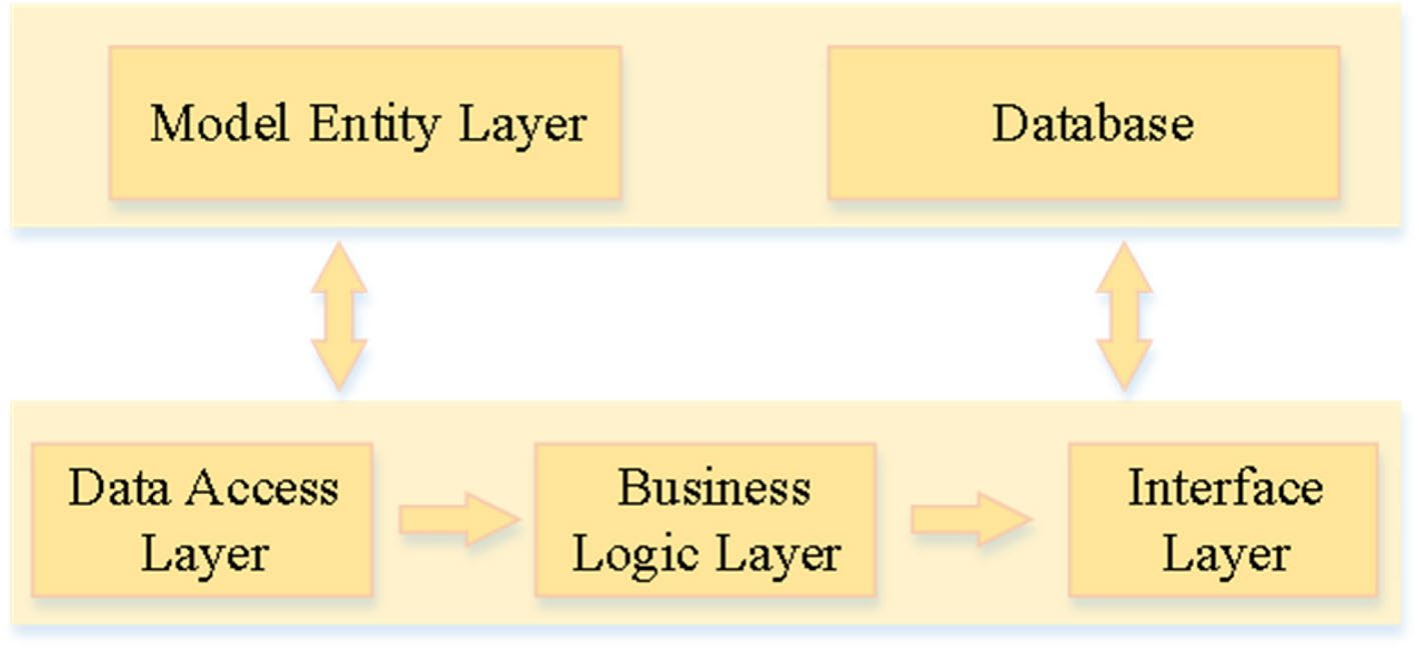
The system architecture

The Django architecture is used in this system, which not only has a clear division of labor, but also does not interfere with each other. The method used in this paper can reduce the coupling of the system, whose architecture is shown in Fig. 2.

This system consists of two modules, one is the management module, and the other is the analysis module. The management module implements student information management and evaluation data management, and the analysis module can not only realize the matching of questionnaire and educational psychology, but also realize the extraction of characteristics and the construction of knowledge graph.

Firstly, the system selects some modules as the initial URL, and then the HTML structure can be viewed through the developer mode. By custom page link parser, you can get other URLs contained in this module, which is then stored in the site URL list and stored locally in text format. When crawling the text, the text judgment mechanism is added in order to eliminate non-ideological and political data. Before storing the text locally, it is necessary to determine whether there are ideological and political terms in the text. If there are no ideological and political terms, no operation is carried out, the system will continue to access other URLs until the URL queue is empty.

The calculation of logical unit is shown in Formulas (5) to (7).

5 $$i = \delta (w_{t} \cdot [h_{t} \,,\,x_{t} ] + d_{t} )$$

6 $$f = \delta (w_{f} \cdot [h_{t} \,,\,x_{t} ] + d_{f} )$$

7 $$o = \delta (w_{o} \cdot [h_{t} \,,\,x_{t} ] + d_{o} )$$

Where $i$ represents the input gate, $f$ represents the output gate, $o$ represents the forget gate, $\delta$ represents the activation function, $d_{t}$、$d_{f}$ and $d_{o}$ represent the offset vector.

### The knowledge storage

The representation forms of knowledge graph are RDF triples and graph database. The RDF can store data in the form of metadata, and the graph database stores and inquires data in the form of graphs. This paper selects RDF triples to represent the ideological and political knowledge triples, and the API is used to store RDF files into the graph database.

**Fig. 3:**
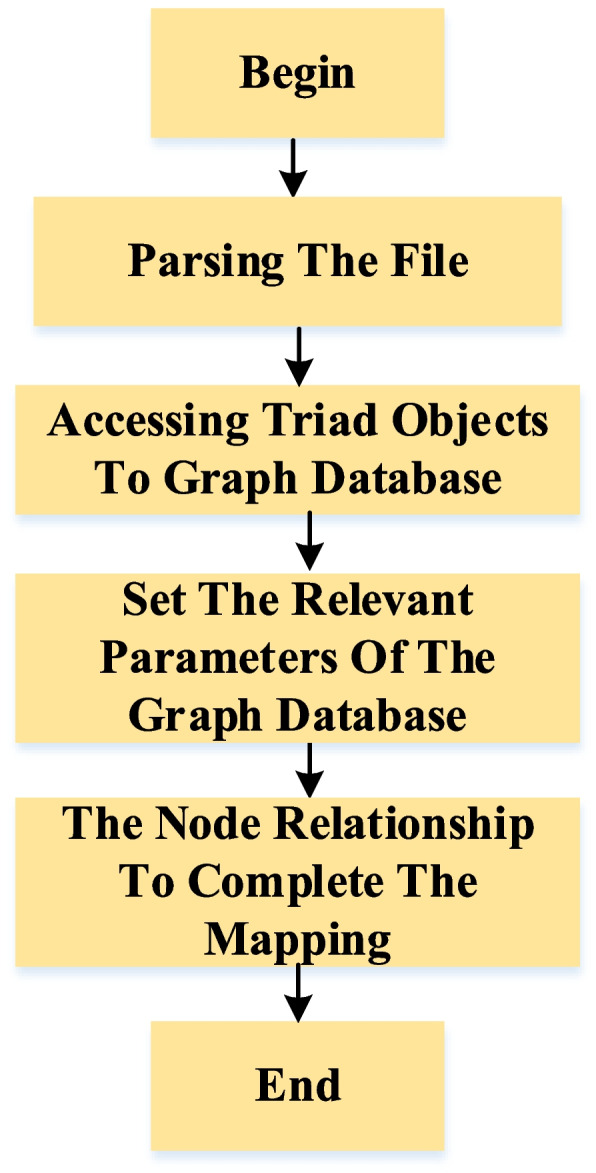
The flow chart of knowledge storage

Then, it is necessary to build a data interpreter in RDF files and graph databases to complete the knowledge storage. Firstly, the API is needed to parse the obtained RDF files and encapsulate the subjects, in which the RDF files are used to encapsulate representation subjects, predicates, and objects. Secondly, the encapsulated triple objects are connected to the graph database, then we need to parse it with a built data parser. Finally, the relevant parameters of the graph database are set, and the API is used to store node relationships in batches inside the server. The flowchart is shown in Fig. 3.

## The analysis of system performance

The relationship prediction system between these two concepts is a complicated system, whose practicability needs to be tested in practice. The knowledge graph is applied to the prediction of the relationship between these two concepts, so the performance of the system should be tested.

### Evaluation index

The effectiveness of the clustering algorithm is measured by Accuracy, Recall and F1 value. Let TP represent the true example, TN represent the true negative example, FP represent the false positive example, FN represent the negative example, then the specific calculation formula of each evaluation index is as follows:

8 $$Precision = \frac{TP + TN}{{TP + FP + FN + TN}}$$

9 $${\text{Re}} call = \frac{TP}{{TP + FN}}$$

10 $$F1 = \frac{2*Precision*Recall}{{Precision + Recall}}$$

### The classification effect of system model

**Fig. 4:**
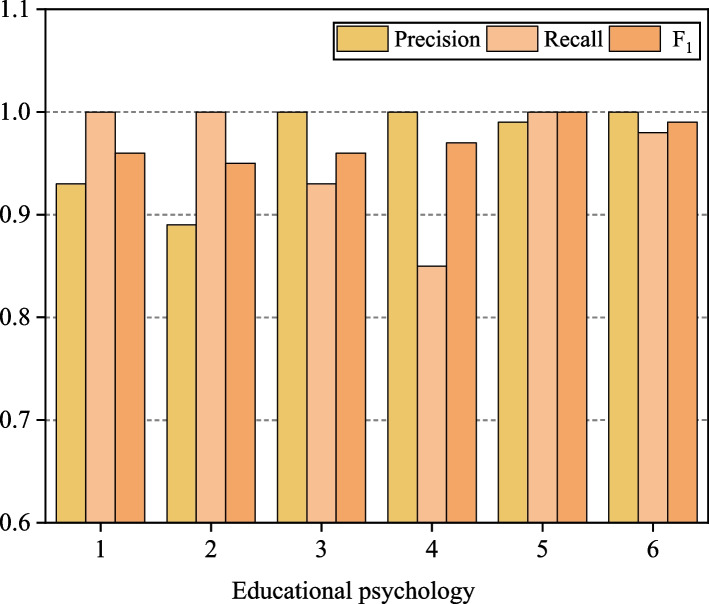
The classification effect of samples

In the relationship prediction system, the text classification effect of the model is of great significance. In this paper, the k-NN model is used to classify the samples, and the classification effect of the classified samples is tested. The corresponding distribution of questionnaire and educational psychology is tested, in which the sample size is 200 and the corresponding distribution of questionnaire and educational psychology can be obtained by k-NN model. The test results of precision, recall and F1 value are shown in Fig. 4.

The assessment of the kNN model reveals its remarkable classification prowess, exemplified by precision, recall, and F1 parameters consistently surpassing 0.95 across diverse educational psychology datasets. These high F1 scores signify the model's exceptional capability in accurately classifying samples. Furthermore, the model demonstrates elevated precision and recall values, indicating its sensitivity to various category datasets. The overall performance of the k-NN model in the relationship prediction system is commendable, showcasing its proficiency in achieving precise sample classification. The model's robust classification effects, as evidenced by its performance metrics, affirm its reliability and effectiveness in accurately predicting relationships within the system.

### The prediction of the relationship

**Fig. 5:**
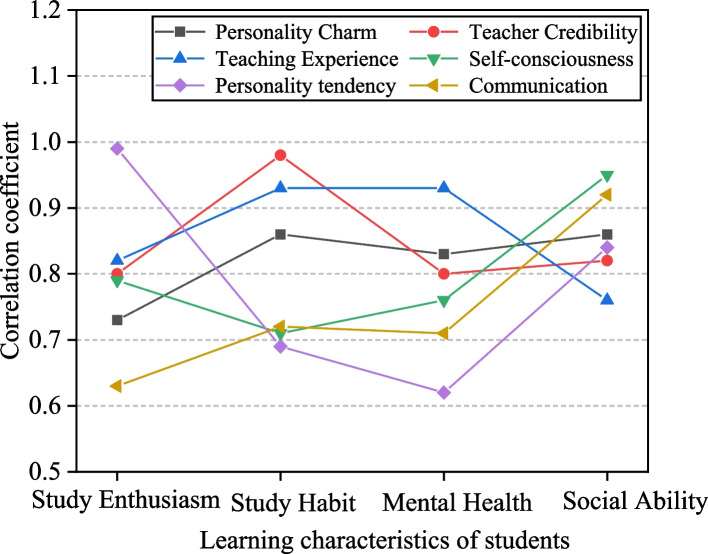
The prediction of the relationship

In order to predict the relationship between different educational psychology and the learning effect, the relationship between different educational psychology and the learning characteristics of students is explored in this paper. The learning characteristics are divided into four levels, which are the learning enthusiasm, the learning habits, the mental health and the social ability. The correlation coefficient is used to characterize the matching degree of different educational psychology and learning characteristics. The greater the correlation coefficient is, the greater the correlation between educational psychology and learning characteristics is. The results are shown in Fig. 5.

**Fig. 6:**
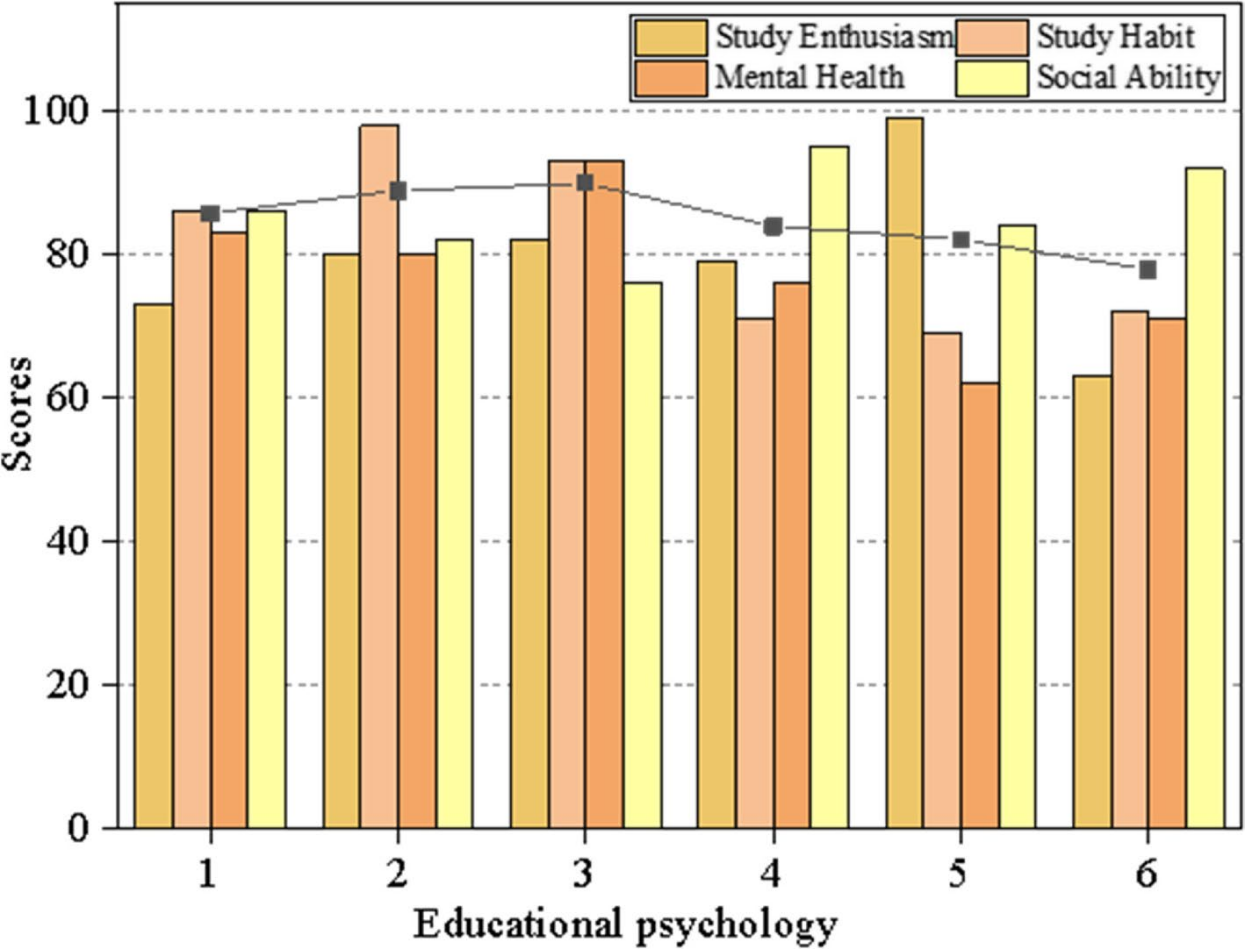
The synthetic evaluation

In Fig. 5, various curves delineate distinct facets of educational psychology, specifically focusing on personality allure, teacher prestige, teaching experience, self-awareness, personality proclivity, and teacher-student communication. A heightened correlation coefficient between educational psychology and learning attributes signifies a stronger association between them. Figure 6 illustrates that the correlation coefficient between personality proclivity and learning enthusiasm is the most substantial, nearing 1. This suggests that personality proclivity significantly enhances enthusiasm for ideological and psychological learning. In contrast, when compared to other psychological factors, the correlation coefficient between political learning habits and teacher prestige is the most pronounced, indicating that teacher prestige plays a pivotal role in fostering ideological and political learning habits.

Furthermore, in comparison to other psychological elements, teaching experience exhibits the highest correlation coefficient with mental health, signifying a robust correlation between teaching experience and mental well-being. Similarly, self-awareness demonstrates the most noticeable correlation with social aptitude. These results underscore the efficacy of the proposed model, showcasing its capability to predict relationships effectively.

To discern the impact of diverse educational psychology on learning attributes, a comprehensive assessment of their effects is undertaken. This approach allows for the identification of the educational psychology exerting the most profound overall influence on learning characteristics. This insight proves invaluable for educators seeking to enhance teaching quality. The effects of various educational psychology factors on distinct learning characteristics are graphically depicted in Fig. 6.

The abscissa represents different educational psychology in the figure, namely the personality charm, the teacher prestige, the teaching experience, the self-consciousness, the personality tendency and the communication between teachers and students. The line chart is the comprehensive evaluation of different educational psychology on learning characteristics. As can be seen from Fig. 6, the teaching experience has the greatest comprehensive impact on the learning characteristics, and followed by the prestige of students. In the teaching, teachers should change teaching strategies accordingly, which can improve the teaching quality.

### The response time of system

**Fig. 7:**
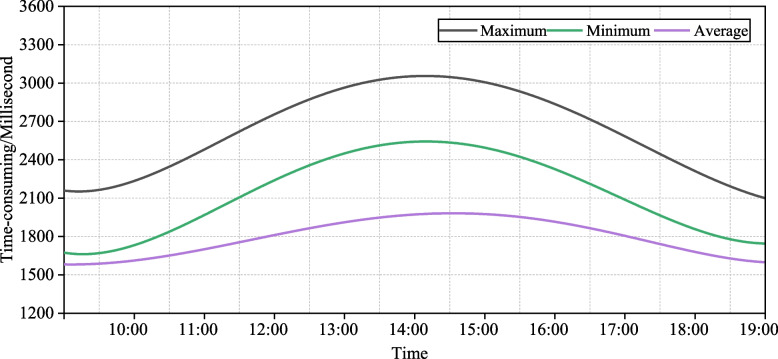
The response time of the system

The response time refers to the time consumed by users when they use the system, which starts from clicking on a page to timing, and ends at this time when the page is completely displayed in the browser. The smaller the response time is, the faster the processing speed of the system is. The response time of the relationship prediction system is very important to the user experience, so the response time of the system is tested in this paper, and the results are shown in Fig. 7.

The response time should take into account the number of users, the more users are, the faster the response time must be. Generally, when the user can get the response within 2 s, the system is sensitive. When the user gets the response within 2 ~ 5 s, the reaction of the system is more sensitive. When the user gets the response within 5 ~ 8 s, the reaction of the system is not very sensitive. The peak value of the maximum response time curve is about 3 s, indicating that the response time of the platform is fast. The peak of the minimum response time curve of the system is only 1.5 s, the response time of the platform is good. The peak value of the average response time curve of the system is about 2.5 s, and the average response time of the system is less than 3 s, which meets the actual needs.

## Discussion

The infusion of psychological principles into the realm of online education charts a transformative course toward personalized and efficacious learning encounters. Through the adept utilization of intelligent technology and knowledge graph-based systems, educators can scrutinize students' psychological profiles, customizing content and interventions to cater to individual needs. This methodology not only heightens the predictability of online education but also confronts mental health challenges by discerning indications of distress and offering timely support. The ongoing progression of intelligent systems in educational psychology holds promise for data-informed decision-making, honing teaching methodologies, and crafting more inclusive online learning environments. Nonetheless, ethical considerations concerning student privacy and well-being must be judiciously navigated to guarantee the responsible and ethical integration of these advancements within the dynamic landscape of online education. Integrating educational psychology into online education holds immense potential for revolutionizing the learning experience and addressing the evolving needs of students. The implications of this integration are multi-faceted and extend beyond the immediate improvements in the relevance and predictability of online education.

### Enhanced personalization

By leveraging educational psychology in online education, personalized learning experiences can be tailored to individual students' cognitive, emotional, and social needs. Intelligent systems can adapt content, pace, and assessment methods to align with students' learning styles and preferences, fostering a more engaging and effective learning environment.

### Improved mental health support

Online education, if not managed properly, can contribute to stress and mental health challenges for students. Integrating educational psychology enables the development of systems that can identify signs of psychological distress and provide timely support. This could include adaptive interventions, counseling resources, or mechanisms for fostering a positive online learning community.

### Data-driven decision making

The knowledge graph-based system described in the paper demonstrates the potential for data-driven decision-making in education. Analyzing psychological characteristics through algorithms and predictive models can offer valuable insights into students' learning patterns, allowing educators to make informed decisions about instructional strategies, interventions, and curriculum development.

### Continuous improvement in teaching strategies

The integration of educational psychology in online education facilitates ongoing assessment and refinement of teaching strategies. By analyzing the psychological mapping of students, educators can adapt their approaches to better suit the diverse needs of their learners, ultimately enhancing the overall quality of education.

### Evolution of intelligent systems

As technology continues to advance, the integration of educational psychology into online education is likely to evolve further. More sophisticated algorithms, machine learning models, and artificial intelligence systems may emerge, providing increasingly accurate and nuanced insights into students' cognitive processes, emotional states, and learning trajectories.

### Global accessibility and inclusivity

The application of educational psychology in online education can contribute to the creation of more inclusive learning environments. By understanding and addressing diverse learning needs, online education can become more accessible to individuals with different abilities, backgrounds, and learning preferences, fostering a truly global and inclusive educational landscape.

### Ethical considerations and privacy

The integration of psychological analysis tools raises ethical concerns related to student privacy and data security. As these technologies advance, it will be crucial to establish robust ethical guidelines, ensuring that the benefits of educational psychology in online education are achieved without compromising the privacy and well-being of students.

## Conclusion

### Theoretical implications

The establishment of the relationship prediction system between educational psychology and ideological and political education holds significant theoretical implications. By presenting a comprehensive model that encompasses the overall framework, questionnaire-psychological mapping, psychological characteristic analysis, and knowledge graph construction, this study contributes to the theoretical foundation of predicting teaching outcomes. The integration of educational psychology with ideological and political education enriches our understanding of the underlying dynamics in pedagogical settings. Theoretical frameworks developed in this paper offer valuable insights for researchers exploring the intersection of psychological principles and ideological education.

### Managerial or policy implications

The practical utility of the developed system extends to managerial and policy considerations within educational institutions. The demonstrated accuracy, recall, and F1 values of the system underscore its potential as a valuable tool for decision-makers in educational settings. Managers and policymakers can leverage the system to enhance teaching predictability, leading to more effective educational strategies. This paper advocates for the adoption of the proposed system in educational institutions, emphasizing its ability to promote the integration of educational psychology and ideological and political teaching. Such integration can have positive ramifications for the overall quality of education.

### Ideas for future research

While this study provides a robust foundation for the relationship prediction system, several avenues for future research emerge. Firstly, further exploration into the fine-tuning of the k-NN model for questionnaire-psychological mapping could enhance the precision of predictions. Additionally, investigations into the scalability of the system across diverse educational contexts and demographics could broaden its applicability. Future research endeavors may delve into refining the clustering algorithm for a more nuanced analysis of psychological characteristics. Furthermore, exploring the long-term impacts of integrating the developed system into educational practices could provide insights into sustained pedagogical improvements. Overall, these suggested areas for future research aim to advance the understanding and practical implementation of predictive models in educational psychology and ideological and political education.

## Data Availability

No datasets were generated or analysed during the current study.

## References

[1] Beresford P (2019). Public participation in health and social care: exploring the co-production of knowledge. Front Sociol.

[2] Patsali ME, Mousa DPV, Papadopoulou EVK (2020). University students’ changes in mental health status and determinants of behavior during the COVID-19 lockdown in Greece. Psychiatry Res.

[3] Wang Y (2020). RETRACTED ARTICLE: Analysis on the construction of ideological and political education system for college students based on mobile artificial intelligence terminal[J]. Soft Comput.

[4] Ma R, Chen X (2023). Intelligent education evaluation mechanism on ideology and politics with 5G: PSO-driven edge computing approach. Wireless Netw.

[5] Hogan A, Blomqvist E, Cochez M (2021). Knowledge graphs. ACM Computing Surveys (CSUR).

[6] Guo Q, Zhuang F, Qin C (2020). A survey on knowledge graph-based recommender systems. IEEE Trans Knowl Data Eng.

[7] Wang H, Zhao M, Xie X. Knowledge graph convolutional networks for recommender systems. The world wide web conference. 2019; 3307–3313.

[8] Wang X, Huang T, Wang D (2021). Learning intents behind interactions with knowledge graph for recommendation. Proc Web Confer.

[9] Huang X (2011). Study of Personalized E-Learning System Based on Knowledge Structural Graph. Procedia Eng.

[10] Yahya M, Breslin JG, Ali MI (2021). Semantic web and knowledge graphs for industry 4.0. Applied Sci.

[11] Liao Y, Huang Q, Wang C. Knowledge graph and its applications in MOOC and SPOC. 2019 2nd International Conference on Contemporary Education and Economic Development (CEED). 2019; 301–305.

[12] Tiddi I, Schlobach S (2022). Knowledge graphs as tools for explainable machine learning: A survey. Artif Intell.

[13] Dewi R, Sitorus Pane B, Lubis AP (2023). THE APPLICATION OF THE GAME METHODS AND INTEREST TO ELEMENTARY SCHOOL STUDENTS’LEARNING OUTCOMES OF FUNDAMENTAL MOVEMENT SKILLS IN RUNNING. Jurnal Halaman Olahraga Nusantara (HON).

[14] Wei X, Saab N, Admiraal W (2023). Do learners share the same perceived learning outcomes in MOOCs? Identifying the role of motivation, perceived learning support, learning engagement, and self-regulated learning strategies. Int Higher Educ.

[15] Frenzel, A. C., Goetz, T., & Stockinger, K. (2024). Emotions and emotion regulation. In Handbook of educational psychology (pp. 219–244). Routledge.

[16] Gojkov-Rajić, A., Šafranj, J., & Gak, D. (2023). Self-Confidence in Metacognitive Processes in L2 Learning. International Journal of Cognitive Research in Science, Engineering & Education (IJCRSEE), 11(1).

[17] Jensen KJ, Mirabelli JF, Kunze AJ, Romanchek TE, Cross KJ (2023). Undergraduate student perceptions of stress and mental health in engineering culture. Int J STEM Educ.

[18] X Deng Practice and Research of Ideological and Political Education Based on Data Mining Technology 2022 11th International conference of information and communication technology (ICTech)) IEEE 2022 287 291

[19] Wu G (2022). The Exploration and Practice of Constructing the Ideological and Political System of" Mechanical Drawing" Course Following" One Body and Two Wings". Int J Soc Sci Educ Res.

[20] Cheng P, Yang L, Niu T (2022). On the ideological and political education of material specialty courses under the background of the internet. J Higher Educ Res.

[21] Luo S (2022). Construction of situational teaching mode in ideological and political classroom based on digital twin technology. Comput Electr Eng.

[22] Hudiyana J, Milla MN, Muluk H (2022). Islam and Politics: A Latent Class Analysis of Indonesian Muslims Based on Political Attitudes and Psychological Determinants. J Soc Polit Psychol.

[23] Salvati M, Giacomantonio M, Pellegrini V (2022). Conspiracy beliefs of Italian voters for populist parties: The moderated mediational role of political interest and ideological attitudes. Acta Physiol (Oxf).

[24] Qiao L (2022). Teaching quality evaluation of ideological and political courses in colleges and universities based on machine learning. J Mathematics.

[25] Alfarshouti AM, Almutairi SM (2022). An Intrusion Detection System in IoT Environment Using KNN and SVM Classifiers. Webology.

[26] Hossain S, Hasan M M, Mehenaz T. Ramifications of Corruption Perception Index: An Exploratory Data Analyses using DBSCAN. Proceedings of the 2nd International Conference on Computing Advancements. 2022; 6–10.

[27] Granitsa Y, Khujayev S. Cluster analysis of regional indicators using DBSCAN algorithm: 025. Dela Press Conference Series: Economics, Business Management. 2022;2022(001):9–9.

[28] Liang X, Luo L, Hu S (2022). Mapping the knowledge frontiers and evolution of decision making based on agent-based modeling. Knowl-Based Syst.

[29] Li Y, Zhou Y, Zhang Y (2022). DKDFN: Domain Knowledge-Guided deep collaborative fusion network for multimodal unitemporal remote sensing land cover classification. ISPRS J Photogramm Remote Sens.

